# Management of Hyaluronic Acid Filler–Induced Refractory Sterile Abscess With Modified Munhoz‐Cavallieri Lavage Protocol: A Case Series

**DOI:** 10.1111/jocd.70435

**Published:** 2025-09-16

**Authors:** Fernanda Cavallieri, Gabriela Munhoz, Laila Klotz de Almeida Balassiano, Anne Kelly Leroy, Maria Fernanda Tembra, Thaissa Bortolato, José Marcos T. Cunha, Renata Bregunci Meyer, Lucia Ribeiro Balsanelli, Marcia Ramos‐e‐Silva

**Affiliations:** ^1^ Cavallieri Clinic Rio de Janeiro Brazil; ^2^ Member of Brazilian College of Radiology and Diagnostic Imaging and of the National Commission of Ultrasound Brazilian College of Radiology and Diagnostic Imaging Rio de Janeiro Brazil; ^3^ Sector of Dermatology and Post‐Graduate Course in Dermatology, University Hospital and School of Medicine Federal University of Rio de Janeiro Rio de Janeiro Brazil; ^4^ Clínica Gabriela Munhoz Rio de Janeiro Brazil; ^5^ Clínica Maria Fernanda Tembra São Paulo Brazil; ^6^ Sector of Dermatology and Post‐Graduate Course in Dermatology Hospital da Polícia Militar de Minas Gerais Belo Horizonte Brazil; ^7^ Private Office Belo Horizonte Brazil; ^8^ Instituto Dermatológico de Curitiba Curitiba Brazil; ^9^ Federal University of Paraná Curitiba Brazil; ^10^ Centro Dermatológico Marcia Ramos‐e‐Silva Rio de Janeiro Brazil

**Keywords:** complications, dermal filler, hyaluronic acid, immunology, patient management, sterile abscess, ultrasonography

## Abstract

**Background:**

The number of cosmetic procedures using hyaluronic acid (HA) has grown significantly in recent years and, consequently, the number of HA injection‐induced complications has also increased. The recognition of sterile abscess as a complication of HA fillers is relatively recent and can evolve to disfigurement. Ultrasonography (USG) examination can be an important tool to diagnose, monitor, and guide therapeutic interventions in filler complication scenarios. Despite the number of complications after filling with HA increasing in scientific publications, especially in the last 5 years, dermatologists are still learning to manage these events and there is no standardized treatment consensus.

**Aims:**

The cases herewith report the successful treatment of HA injection‐induced refractory facial sterile abscess, including the safety of high doses of hyaluronidase.

**Patients/Methods:**

Four adult women with refractory facial sterile abscesses due to HA filler were treated with high doses of hyaluronidase (range: 22 500–258 000 IU) by modified Munhoz‐Cavallieri lavage protocol after unsuccessful treatment, including antimicrobials, corticosteroids, and simple drainage.

**Results:**

All patients required two or more lavages for resolution, with a minimum interval of 48 h and presented complete resolution of the refractory sterile abscesses with no relapse.

**Conclusion:**

The cases herewith emphasize the importance of recognizing and correctly managing sterile abscesses to determine a favorable outcome during clinical practice, avoiding prolonged and ineffective treatment, as well as the relevance of USG to the diagnosis, guidance of intervention, and treatment monitoring of filler complications.

## 1 Introduction

The number of cosmetic procedures using hyaluronic acid (HA) has grown significantly in recent years [1]. The relative safety of HA products as dermal fillers is due to their biocompatibility, effectiveness, versatility of application, good stability, and reversibility by hyaluronidase [2].

However, many HA‐based products present different rheologies and cross‐linkage degrees to stabilize and extend their effects [3, 4]. This fact, combined with numerous aesthetic procedures performed by nonmedical and untrained professionals, may be related to the increase in complication rates [4, 5, 6, 7, 8].

The description of sterile abscess as a complication of HA filler is relatively recent. The pathogenesis is unclear, although some hypotheses consider that the immune system is involved [1, 6]. Despite the number of complications after filling with HA increasing in scientific publications, especially in the last 5 years, dermatologists are still learning to manage these events, and there is no standardized treatment consensus [1, 6].

In a complication scenario, the ultrasonography (USG) examination appears as an important tool to identify cosmetic fillers, as well as to diagnose, monitor, and guide therapeutic interventions in filler complication scenario [9].

In this report, we describe four cases of refractory facial sterile abscesses after filling with HA that were completely resolved only with the modified Munhoz‐Cavallieri lavage protocol (mMCP) despite the previous treatment received that was not initially responsive.

## 2 Material and Methods

Four adult women with refractory facial sterile abscesses due to HA filler were treated by our group with mMCP until complete resolution from 2022 to 2024. All patients provided consent for the use of their medical information and images in scientific publications.

Demographic and clinical data of these patients are presented in Table 1.

**TABLE 1:** Demographic and clinical data of four patients with facial refractory sterile abscess induced by HA‐filler.

Patient	Age	Sex	Brands of HA	Site of HA injection	Dose of HA	Onset of symptoms after HA injection	Trigger	Signs and symptoms	Laboratory exams	Site of sterile abscess	Treatment before the mMCP procedure	Hyaluronidase dose	Number of mMCP	Complete resolution?
1	70	Female	Restylane Lift	Mentolabial fold	2 mL	10 days	No	Bilateral edema associated with phlogistic signs at the injection sites	Normal leukocytosis ESR and CRP Negative Culture	Mentolabial fold	3 months of oral treatment, antimicrobials and oral corticosteroids, including methotrexate (MTX) and cyclophosphamide, and two surgical interventions	7500 IU/session Total: 22 500 IU	3	Yes
2	46	Female	Rennova Lift Plus	Mandibular arch, chin and mentolabial fold	5 mL	60 days	No	Edema in the right mentolabial fold	Normal leukocytosis ESR and CRP Negative culture	Right mentolabial fold and submental space	4 months of oral treatment, antimicrobials and oral corticosteroids	4 sessions: 29 625 IU/session (3 solutions—2× 10 500 IU + 1× 8625 UI) 4 sessions: 34 875 IU/session (3 solutions—2× 12 000 IU + 1× 10 875 IU) Total: 258 000 IU	8	Yes
			Restylane Volyme	Zygoma	1 mL									
3	40	Female	Juvederm Volift Juvederm Voluma	Temporal, zygomatic, preauricular, chin, and mandibular arch	8 mL	10 days	Chondritis	Cervical and left retro auricular tenderness, fever, asthenia, myalgia, and the left auricle was notably edematous	Mild leukocytosis and elevated CRP Negative culture	Zygomatic, preauricular and mandibular arch	Oral and IV antibiotics Oral corticosteroids	1 session: 24 000 IU (2 solutions 12 000 IU) 2 sessions: 23 000 IU (2 solutions—1× 12 000 IU + 1× 11 000 IU) Total: 70 000 IU	3	Yes
4	57	Female	Hialurox	Malar, mandibular line, chin, pre jowl and lips	8 mL	180 days	No	Intense neck and facial pain accompanied by erythema and edema	Normal leukocytosis ESR and CRP Negative culture	Temporal, mandibular, malar and nasolabial fold	Oral and IV, antimicrobials and corticosteroids	1st session: 33 000 IU (3 solutions—1× 12 000 IU + 2× 10 500 IU) 2nd session: 16 000 IU (2 solutions—9000 IU + 7000 IU) Total: 46 000 IU	2	Yes
			Perfectha	Malar and mandibular line	4 mL									

The sterile abscess was determined based on exclusion diagnosis that includes (1) clinical signs and symptoms, (2) blood test, (3) USG assessment, (4) previous unsuccessful treatment, and (4) aspirated fluid with negative cultures.

All patients presented palpable nodules with mild phlogistic signs. The blood test showed normal or mild leukocytosis, and mild or no increase in the erythrocyte sedimentation rate (ESR) and C‐reactive protein (CRP).

All of them had received unsuccessful treatment prior to attending the clinic, including antimicrobials, corticosteroids, and/or simple drainage.

After clinical history, the mMCP was implemented, which consists of three steps: including facial USG (1) to assess the presence of a collection, (2) to guide the fluid collection aspiration, and (3) to guide the lavage protocol.

USG images were obtained with EPIQ Elite equipment (Philips Medical Systems, Bothell, WA, USA), and a high frequency linear transducer (4–18 MHz) was used, employing a B‐mode assessment associated with Color Doppler to evaluate local vascularization. A large volume of gel was used between the transducer and the region, and the least possible compression with the transducer was applied to obtain the best accuracy of superficial structures. A complete facial mapping was conducted in all patients, including not only clinically affected areas but also unaffected regions, allowing for full‐face assessment.

The USG criteria for abscess detection included the presence of a well‐defined collection with heterogeneous or thick internal content, frequently exhibiting anechoic to hypoechoic areas with internal debris, suggesting high proteinaceous material on B‐mode. On color Doppler imaging, the most prevalent finding was mild peripheral vascularization. The size of the abscesses ranged from 4 mm to 3.5 cm, and in all cases, they were situated in the subcutaneous plane.

Due to the presence of the collection, US‐guided drainage was performed using a 21G × 1¼″ or 18G × ½″ needle attached to a 5 or 10 mL empty syringe into the abscess cavity, followed by aspiration for 5 s or until fluid is retrieved. The patients were not using antibiotics and/or oral corticosteroids at the time of the drainage. Considering that the pre‐analytical process is of great importance for reliable results, a sterile gel and sterile probe cover were applied to maintain aseptic conditions during the procedure in compliance with the microbiologist's recommendation. Anesthetic button with lidocaine was performed to allow the needle entry hole. Needle visualization was performed using the in‐plane technique in all cases, allowing real‐time observation of the entire needle trajectory and confirmation of the needle tip within the collection prior to the initiation of lavage.

Aspirated fluid was sent immediately for microbiologic analysis, including microscopy of a thin smear stained with Gram and Ziehl–Neelsen stains and culture for typical and atypical bacteria as well as for mycobacteria. There was no growth of microorganisms in any case, and therefore the authors proposed the term “sterile abscess” for this specific complication.

After collection drainage, each patient was then treated with the lavage solution that consists of high doses of hyaluronidase (range: 4500–12 000 IU) diluted with 6–8 mL of 0.9% saline solution + 1 mL triamcinolone 20 mg/mL + 1–3 mL of 2% lidocaine for a total of 10 mL of solution. Carrying out the washing with a 3 mL syringe and a 22G or 23G needle: injection of 1–3 mL of the solution, aspirating, and disposing. The process is repeated two to three times to wash the collection area. Discard the syringes and needles used in each step. During the last injection, with sterile syringes and needles, 0.5–3 mL of the solution was deposited into the cavity to occupy the purulent secretion space, that is, to collapse the space visualized on the USG and dilute the remaining HA. The protocol can be repeated after 48 h if the collection persists in USG images.

### 2.1 Case 1

**FIGURE 1:**
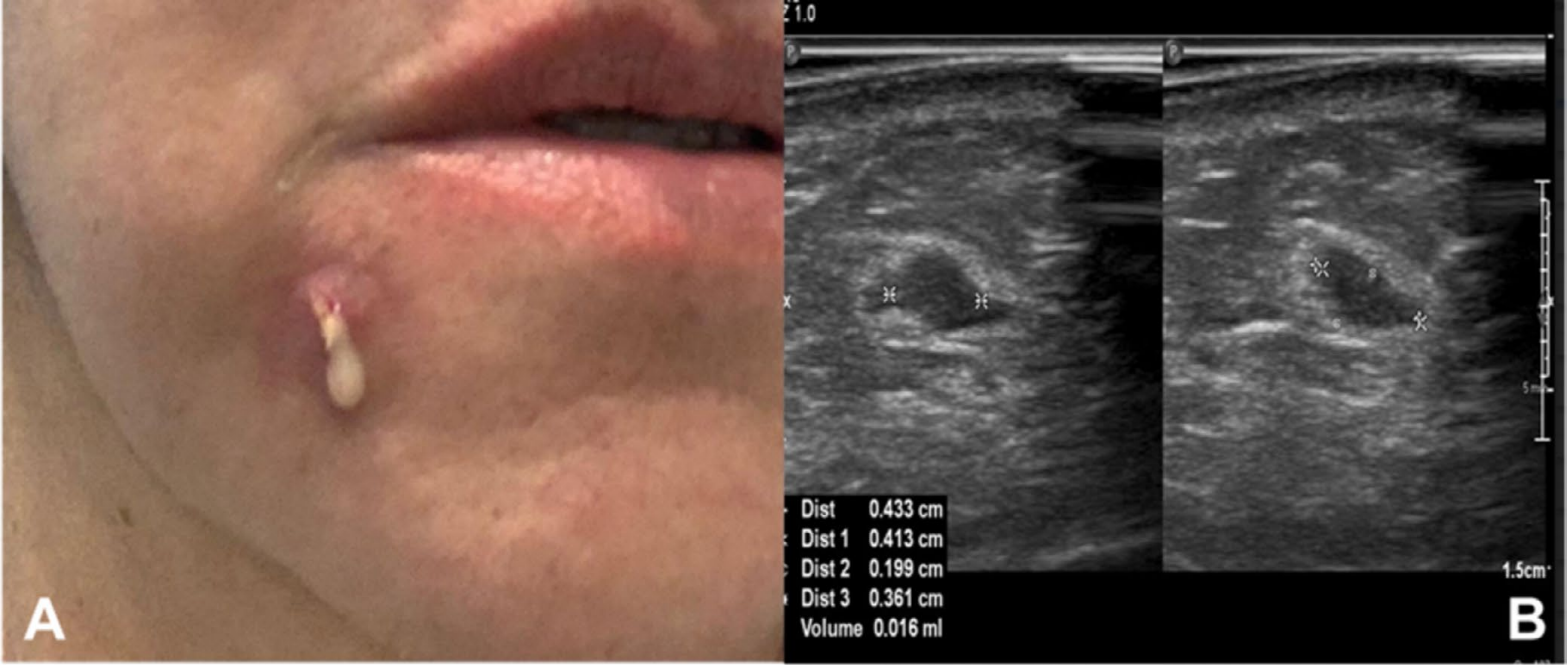
(A) A 70‐year‐old woman with a persistent nodule with spontaneous discharge of pus in the mentolabial fold filled with HA even though 3 months of oral treatment (antibiotics and corticosteroids) and two surgical interventions to treat this HA‐filler complication; (B) B‐mode USG images showing two small thick‐fluid collections in the subcutaneous plane.

A 70‐year‐old woman with dental caries had the mentolabial fold filled with HA and presented bilateral edema associated with phlogistic signs at the injection sites 10 days after the procedure. Prior to attending our group, the patient was treated with different antimicrobials and oral corticosteroids, including methotrexate (MTX) and cyclophosphamide, and drainage by oral mucosa. Even after these treatments, the condition worsened with persistent purulent secretion, and the USG images revealed bilateral fluid collection associated with edema. The patient was submitted to new drainage, and the aspirated fluid obtained with an aseptic technique was sent for cytologic and microbiologic analysis, which revealed the presence of polymorphonuclear cells and no pathogen, respectively. Despite the 3 months of oral treatment and two surgical interventions, the condition did not improve, maintained a nodule and it evolved into spontaneous drainage of pus (Figure 1A), and the patient was referred to our group. A new USG exam showed numerous loculated small fluid collections (Figure 1B). Three sessions of the mMCP guided by USG were performed at 72‐h intervals, totaling 22 500 IU of hyaluronidase. One week after the last session, no fluid collection was observed, and the subcutaneous tissue presented normal USG characteristics. The patient was discharged and followed up for 3 years without relapse.

### 2.2 Case 2

**FIGURE 2:**
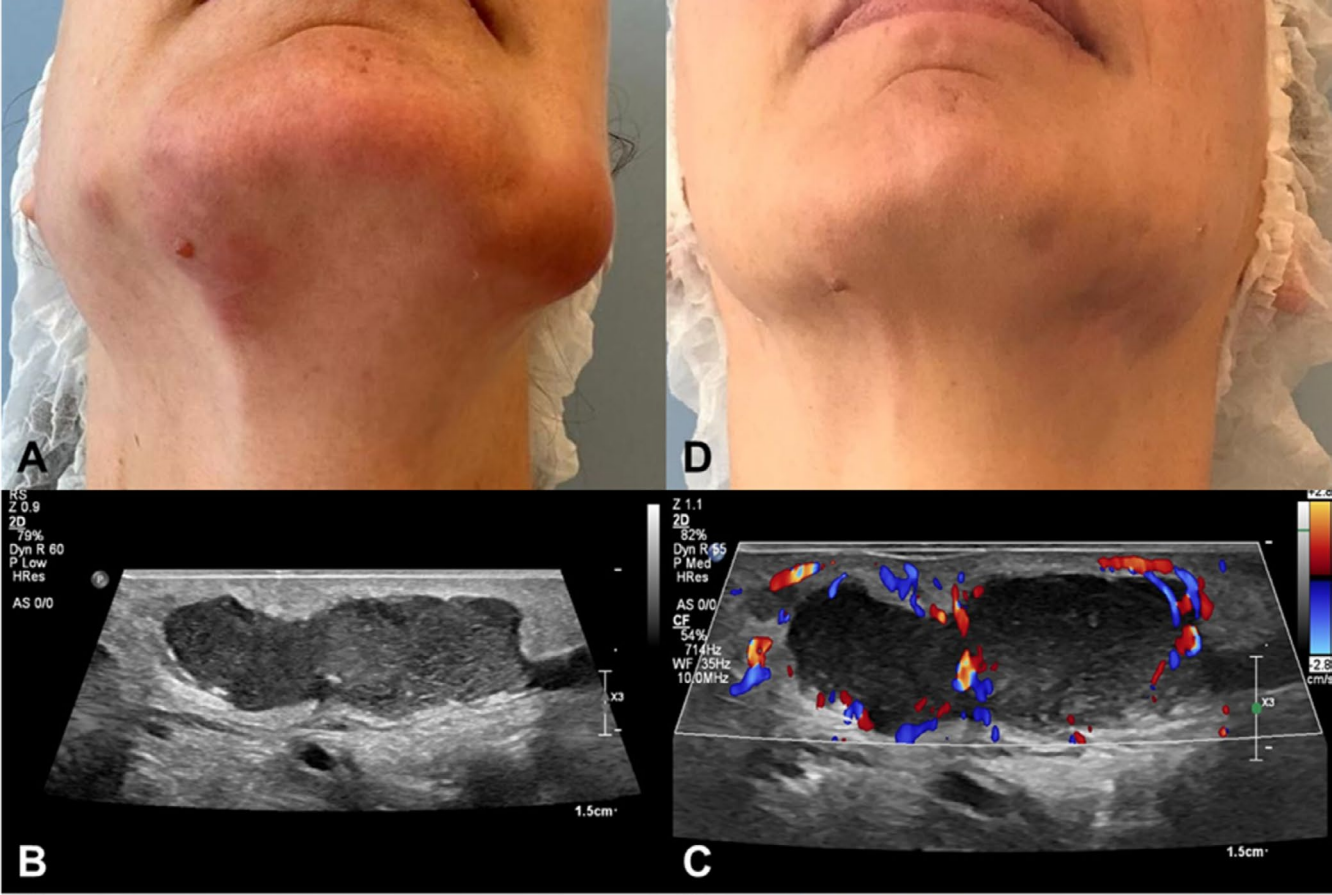
(A) A 46‐year‐old woman with a persistent HA‐filler complication—including nodules and edema in the mandibular region, jowl and preauricular area—even though 4 months of oral treatment (antibiotics and corticosteroids); (B) B‐mode USG image showing the abscess identified by thick fluid collection with debris in suspension, well‐defined borders, and lobulated contours in the subcutaneous plane; (C) image with color Doppler demonstrating the presence of peripheral vascularization around the thick fluid collection (abscess); (D) Clinical follow‐up photograph of the patient 18 months after “Modified Munhoz–Cavallieri” lavage protocol.

A 46‐year‐old woman treated with HA filler in the mandibular arch, chin, mentolabial folds, and zygomatic area presented edema in the right mentolabial fold two months after the procedure. USG images demonstrated a diffuse increase in the thickness and echogenicity of the subcutaneous tissue (panniculitis). Prior to attending our group, the patient was treated with oral antibiotics and corticosteroids for 4 months with no improvement. The edema worsened and progressed to the submental area, and the patient was referred to our group. Clinically, the patient presented nodules and edema in the mandibular region, jowl, and preauricular area (Figure 2A). All medications were discontinued 48 h before the new USG. The USG images showed a large amount of thickened fluid collection, with debris in suspension, affecting the mandibular area and jowl, in addition to the migration of HA to the submental area (Figure 2B) The color Doppler demonstrated increased vascularization around the fluid collection (Figure 2C). Three microbiological tests were performed, all negative. Based on clinical signs and the USG images, our group proposed the mMCP guided by USG. Over a 10‐day period, four lavage sessions were performed with a total of 118 500 IU of hyaluronidase administered. The nodules were still present but with reduced size. After 30 days, the patient underwent an additional four lavage sessions at 48‐h intervals, totaling 258 000 IU of hyaluronidase. Eighteen months post‐intervention, the nodules were completely resolved, and no recurrence of lesions was observed (Figure 2D).

### 2.3 Case 3

**FIGURE 3:**
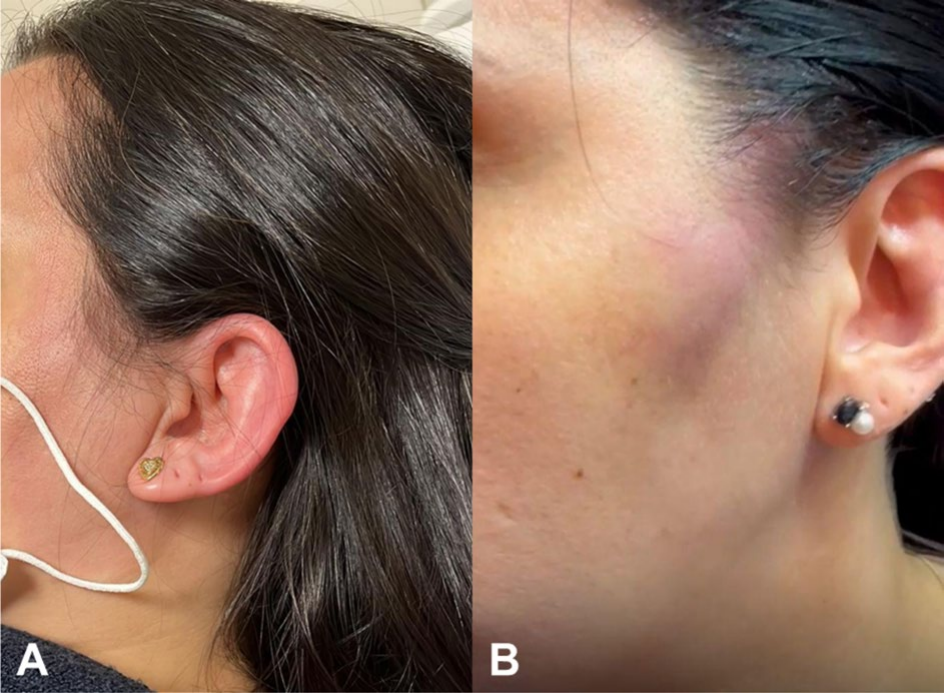
(A) A 40‐year‐old lactating woman presenting a preauricular erythema and intense edema and erythema of the entire left ear region, sparing the earlobe, after Ha filler. The hypothesis is a chondritis after infection in an immunocompromised patient. The chondritis, with its inflammatory condition, was probably the trigger for the sterile abscess in the left zygomatic area (B).

A 40‐year‐old lactating woman filled the temporal, zygomatic, preauricular, chin, and mandibular arch regions with HA. Previously, the patient was treated for 15 days with oral antibiotics and corticosteroids for a respiratory infection. After 10 days of the HA procedure, the patient presented with cervical and left retroauricular tenderness, fever, asthenia, myalgia, and the left auricle was notably edematous. Due to the continuous fever and asthenia, the patient sought hospital care. During the initial evaluation, infectious causes were ruled out. Laboratory tests revealed mild leukocytosis and elevated CRP, which were more compatible with corticosteroid‐induced alterations in inflammatory markers than with an active bacterial infection. The patient was admitted for intravenous antibiotic therapy, considering a facial cellulitis diagnosis. After 4 days, she was discharged with venous antibiotics in home care. Ten days after the onset of the condition, the patient developed an erythematous, painful swelling in the left zygomatic area. The USG images showed a fluid collection in the affected area, as well as a smaller collection in the mandibular angle. At this point, the patient was under the care of our group. We disagreed with the diagnosis of facial cellulitis and raised the hypothesis of chondritis after infection in an immunocompromised patient (Figure 3A). The chondritis, with its inflammatory condition, was probably the trigger for the sterile abscess in the left zygomatic area (Figure 3B). All medications were discontinued 48 h before the mMCP. Microbiological tests were negative for bacteria. Three sessions of the mMCP were performed at 48‐h intervals, totaling 70 000 IU of hyaluronidase, resulting in complete resolution of the condition. The patient was discharged and followed up for 13 months without relapse.

### 2.4 Case 4

**FIGURE 4:**
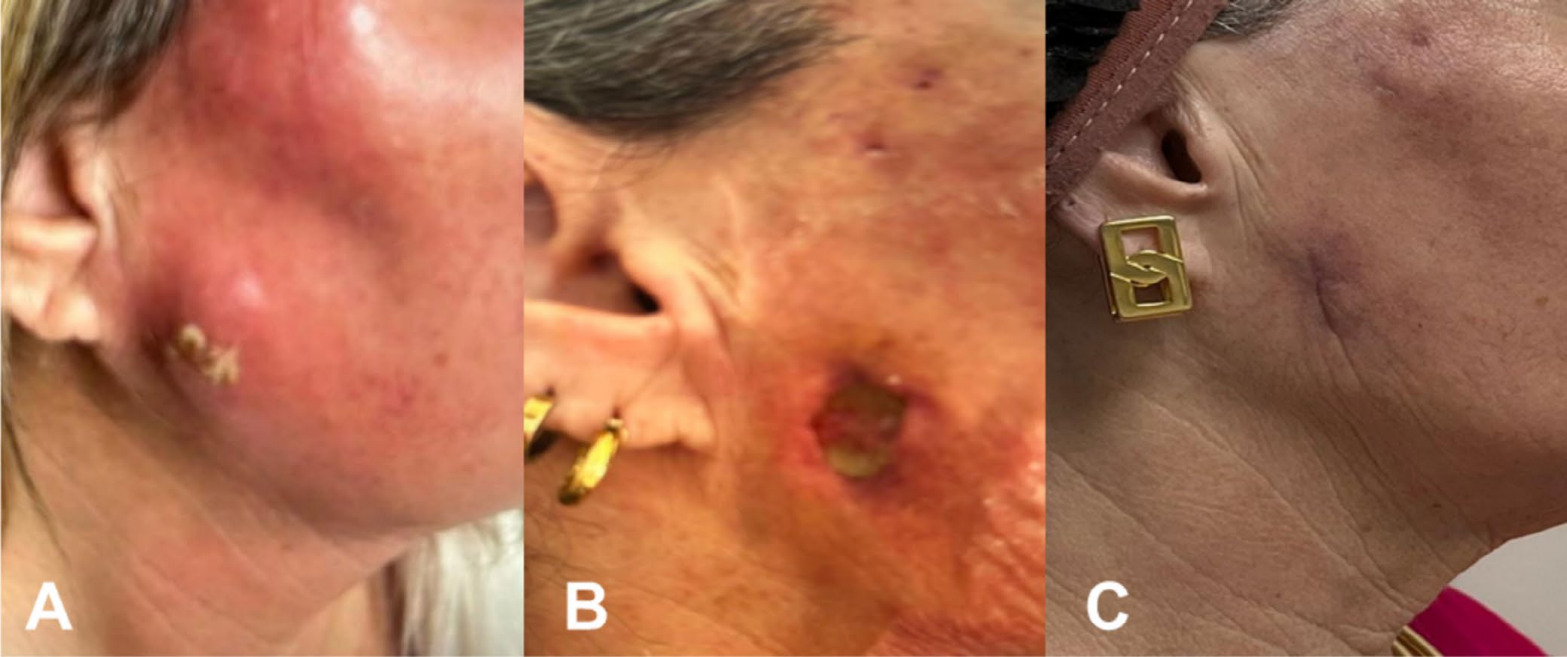
(A) A 57‐year‐old woman treated with several HA injections was hospitalized with extensive edema and erythema of the entire right hemiface, with an area corresponding to the right mandibular angle, and spontaneous pus drainage; (B) Image after surgical intervention demonstrating an ulcerated lesion with spontaneous pus drainage at the level of the right mandibular angle; (C) Complete resolution 9 months after two sessions of the mMCP.

**FIGURE 5:**
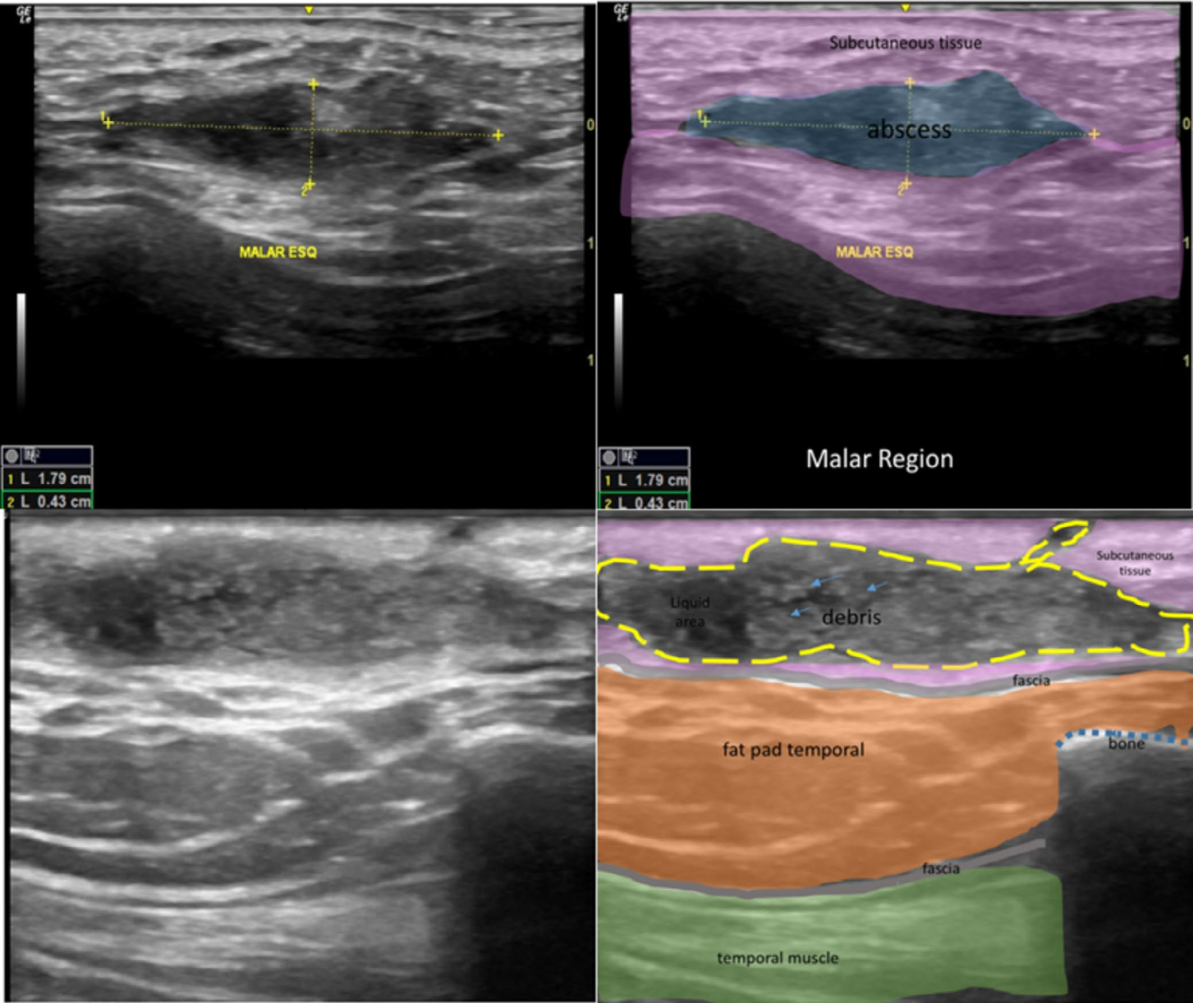
USG images demonstrating two thick fluid collections (abscesses) in the malar and temporal regions. Comparative view: Ultrasound image on the left and annotated image on the right.

A 57‐year‐old woman treated with several HA injections in the malar, mandibular arch, chin, pre‐jowl, and lips presented intense neck and facial pain accompanied by erythema and edema six months after the filler procedure. She received oral treatment with antibiotics and corticosteroids, but the symptoms worsened and she was hospitalized for intravenous antibiotic therapy (Figure 4A). The patient developed a fistula and extensive purulent secretion in the right mandibular arch and underwent surgical intervention with the hypothesis of necrotizing fasciitis (Figure 4B). Due to disfiguring edema, she was referred to the Intensive Care Unit (ICU) for intravenous antibiotics and corticosteroids. Preemptive endotracheal intubation with mechanical ventilation was adopted for 3 days due to impending upper airway obstruction. The patient was discharged and referred to our group. The USG images revealed several fluid collections in the temporal, mandibular, and malar areas (Figure 5) Microbiological tests of the aspirated material for common bacteria and mycobacteria were negative, confirming the diagnosis of sterile abscesses. The mMCP was performed using 33 000 IU of hyaluronidase in the first session. The USG after 72 h revealed small abscesses with no symptoms. After 3 weeks, the patient complained again of mild edema and pain in the cheeks. The USG images showed 3 minor abscesses on the right cheek, right nasolabial fold, and left zygomatic arch. The mMCP was performed again using 16 000 IU of hyaluronidase, resulting in a complete resolution of the condition (Figure 4C). No relapse was observed during the 9 months of follow‐up.

## 3 Results

The refractory sterile abscesses reported in women aged 40–70 years who received different brands of HA for filling the face.

Two patients had early‐onset 10 days after the injection, while the others had developed abscesses 60 days after receiving the filler. Only one patient had partial and transient improvement after receiving intravenous antimicrobial. Three patients worsened despite the various treatments, including repeated drainage, antimicrobials, and corticosteroids. Two patients were hospitalized to receive intravenous medications, but only one was referred to the ICU with preemptive endotracheal intubation for 3 days due to impending upper airway obstruction.

The USG images revealed a thickened liquid collection in all patients, demonstrating the formation of an abscess at the edematous area. Microbiological exams were all negative, confirming the diagnosis of a sterile abscess.

**FIGURE 6:**
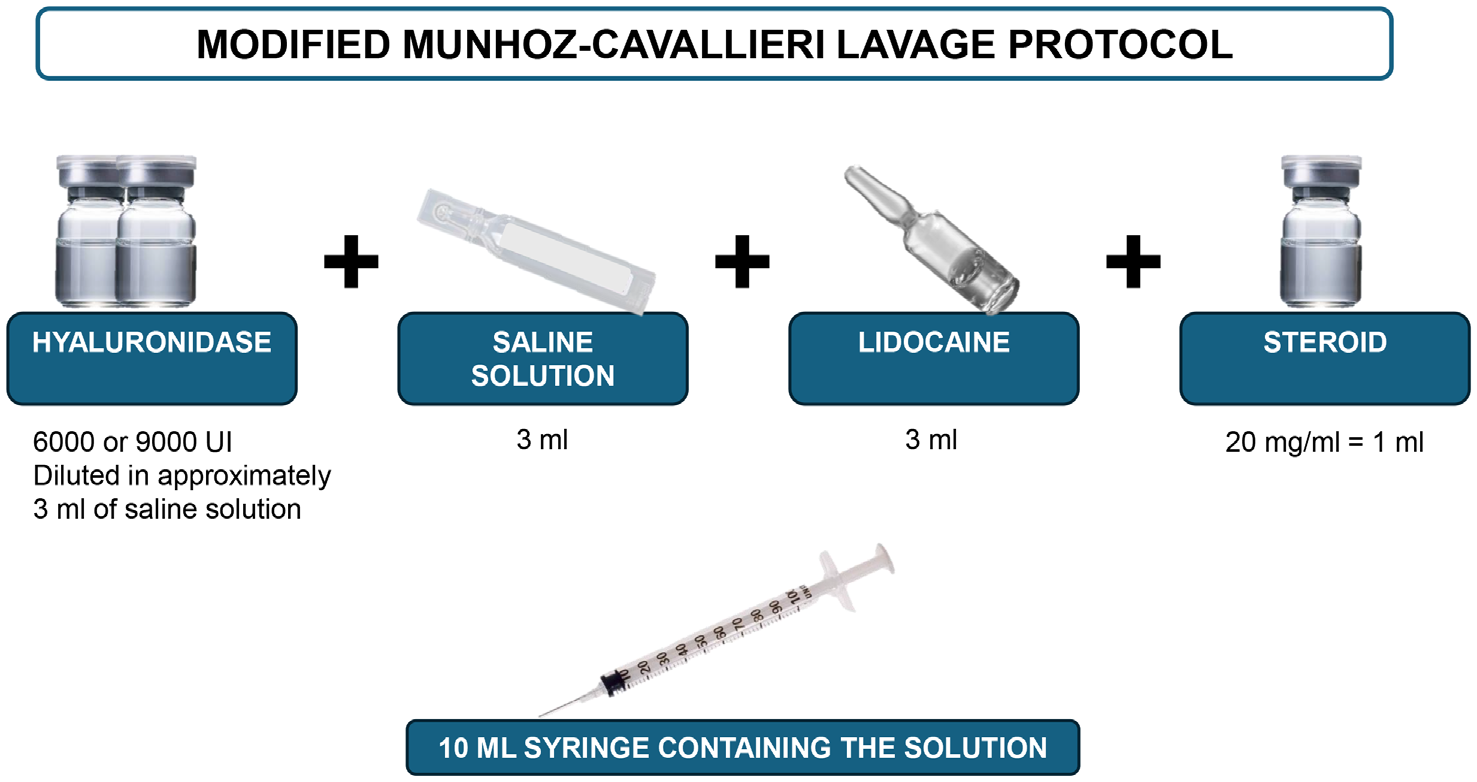
Illustration of modified Munhoz Cavallieri lavage protocol solution.

Due to therapeutic failures, the patients were referred for specialized evaluation and were treated with mMCP (with high doses of hyaluronidase, sterile saline solution, and injectable corticosteroids) (Figure 6).

**FIGURE 7:**
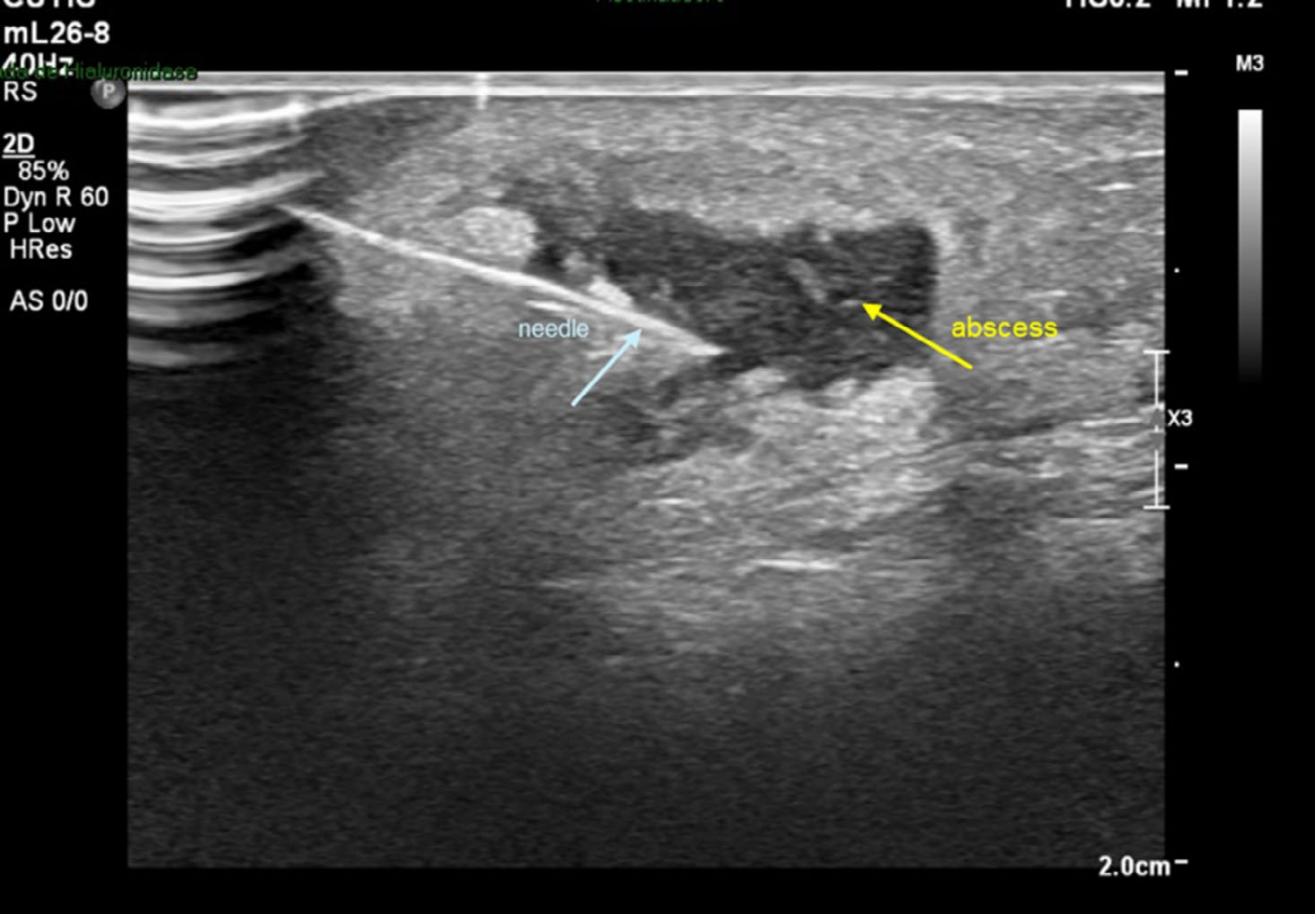
Ultrasound image demonstrating the needle inside the abscess during the mMCP.

All patients required two or more lavages for resolution, with a minimum interval of 48 h and presented complete resolution of the refractory sterile abscesses with no relapse. The success of mMCP depends on injecting the solutions inside the collection; that is why this procedure must be guided by USG (Figure 7).

No adverse events were observed following mMCP treatment (8 months to 3 years after the procedure). The current assessment is based on clinical signs and symptoms.

## 4 Discussion

The sterile abscess in our patients is a poorly known and underreported problem among HA filling developments [1].

Many of these patients are treated as if they have infectious abscesses solely because of the presence of purulent exudate [5, 6].

Inflammatory responses triggered by infections closely resemble those caused by sterile stimuli. Both involve the recruitment of neutrophils and macrophages, the release of inflammatory cytokines and chemokines, and the activation of T cell‐mediated adaptive immunity. This similarity suggests that infectious and sterile triggers may engage common receptors and signaling pathways [10]. Therefore, the purulent secretion in a sterile abscess may be just the result of degraded inflammatory cells, particularly neutrophils, instead of an infection [1].

The sterile abscess as a possible HA filler complication was reported by Tezel and Fredrerikssom in 2007. According to these authors, the body might reject it, initiating undesired reactions such as encapsulation of the gel implant and formation of granule or a sterile abscess [11].

The pathogenesis of HA‐induced sterile abscesses remains unclear, and different hypotheses suggest inflammatory responses due to foreign body reactions, delayed‐type hypersensitivity (Type IV) and biofilm formation [12, 13, 14, 15, 16, 17].

Immune microenvironment of different skin and subcutaneous tissue layers, the injection technique itself, the products' rheology, properties, and degree of crosslinking of HA filler, as well as the presence of preservatives in the product can induce an immune reaction and local inflammatory response [12, 13]. The neutrophils attempted to degrade biomaterial through phagocytosis and activated an inflammatory cascade leading to delayed‐onset complications such as (low‐grade) inflammatory nodules and abscess formation [12].

Although most studies show a decreased inflammatory reaction after several months, it depends on the type of filler used, whereas material from acute lesions (up to 30 days) shows a cellular infiltrate composed mainly of neutrophils, lymphocytes, macrophages, and cells similar to fibroblasts. Tissues from 6 months on show predominantly the presence of small and round empty spaces surrounded by dense and organized inflammatory and fibrous tissue [12].

Multiple cases indicated that patient‐specific factors such as an infectious process (sinusitis, urinary tract infection, respiratory tract infection, dental infection), trauma on the face, or vaccination may contribute to triggering a localized reaction at the site of HA injection [12, 14, 15]. In 2017, Cavallieri et al. [15] described for the first time the Persistent, Intermittent Delayed Swelling (PIDS) in HA injection sites, of which 36% of the cases were triggered by an infectious condition.

In the case of the lactating patient, lactation itself would not serve as a trigger, as this physiological state is typically associated with relative immunosuppression. The sterile abscess was likely secondary to chondritis, which in turn was triggered by a preceding respiratory tract infection.

Delayed hypersensitivity reactions are also often proposed in the literature as causes of HA‐filler complications, which hypothesize that adverse effects could have developed memory T lymphocytes able to start a specific immune response against the HA‐filler [12, 16, 17].

Some authors have proposed that bacterial biofilms—communities of unicellular organisms adhered to a surface via a polysaccharide matrix—may play a role in the pathogenesis of delayed complications related to injectable fillers, as biofilms can result in negative culture findings during laboratory investigations [12, 17]. In cases involving biofilms, treatment typically includes the removal of infected devices and the use of antibiotics with adequate tissue penetration and activity against the implicated organisms [17]. Nevertheless, all patients achieved complete resolution without the use of antibiotics. This supports the hypothesis that the inflammatory reaction observed in these four cases represents an exaggerated foreign body immune response to HA—rheological properties and the larger volume—rather than a bacterial infection. Although this hypothesis is challenging to confirm due to diagnostic limitations, further studies are necessary to understand the exact pathogenesis, mechanism, and prevention of this phenomenon.

Faced with a patient presenting unilateral or bilateral edema with few or no inflammatory signs in a site previously treated with injectable HA, the procedure initially consists of performing an USG exam to assess the presence of a fluid collection [1]. The dynamic visualization of fluid collection with USG allows the aspiration of the material for cytology and microbiological analysis, including microscopy of a thin smear stained with Gram and Ziehl–Neelsen stains and culture for typical and atypical bacteria as well as for mycobacteria. The sterile abscess diagnosis is based on the association of USG images, clinical signs and symptoms, and the laboratory tests which must present negative results for microbiological tests [1, 6].

Despite the number of sterile abscesses having increased, the scientific literature presents few publications of HA‐induced sterile abscesses, and there is no standardized treatment consensus to manage these new events as new complications emerge [18]. André [19] reported in 2004 three cases of sterile abscesses that were treated with antibiotics and corticosteroids; however, only two had complete resolution; one case persisted despite various treatments. In 2013, Rodrigues‐Barata and Camacho‐Martinez [20] reported a possible case of sterile abscess in a patient after HA filler, since the patient presented a recurrent granulomatous reaction with a negative microbiological culture, although they were treated with corticosteroid and antibiotics. Shin et al. [21] reported a sterile abscess that occurred 5 years after HA filler injection to the glabella, which was completely healed after treatment with antibiotics, corticosteroids, and aspiration, even though the microbiological tests showed negative results. von Csiky‐Sessoms [22] reported in 2020 the incision and drainage in association with antibiotics to treat the sterile abscess induced by a permanent polyacrylamide injection that appeared 2 years after the procedure. In 2022, the Munhoz‐Cavallieri lavage protocol (MCP) was proposed by our group based on the successful treatment of eight cases of sterile abscesses after HA injection [1] and in 2023, the modification of this protocol was presented after the total resolution of facial recalcitrant sterile abscesses in five patients that were treated with Juvederm Volux (Allergan) [6].

Some recent articles on complications due to HA may be a case of undiagnosed sterile abscess, as the delayed hypersensitivity reaction type 4 to HA filling triggered by the mRNA vaccine against COVID‐19 described by Azzouz et al. in 2023 [23] and one of the three cases described by Owczarczyk‐Saczonek and De Boulle [24] as ASIA syndrome after HA filling. The patient history shows that multiple courses of antibiotics and/or corticosteroids were ineffective treatments.

Although some cases of sterile abscesses described in the literature have responded to treatment with antibiotics and corticosteroids, our clinical experience has shown that such therapies—including high‐dose regimens—are largely ineffective [19, 20, 21, 22, 23, 24]. The cases herewith reinforce that the use of antibiotics in cases of sterile abscess may prolong the clinical course and have been proven ineffective. Due to this, our clinical practice suggests that the antibiotics should be restricted to confirmed infectious abscesses.

In this context, the MCP has emerged as a promising therapeutic option, offering greater precision and efficiency in the management of sterile abscesses. The combination of hyaluronidase with intralesional corticosteroid therapy brought a favorable outcome by eliminating the trigger (intact filler and its degradation products) and locally inhibiting the inflammatory cells. USG‐guided dissolution is a useful tool to improve the accuracy of hyaluronidase injection into the abscess and its follow‐up until complete resolution. However, more studies are necessary to reach accurate information [1, 6].

The main difference between the original and modified MCP consists of the high doses of hyaluronidase (Table 2). The MCP involves washing the cavity with a lavage solution many times, including common doses of hyaluronidase, sterile saline solution, and injectable corticosteroid, while the mMCP solution was prepared with high doses of hyaluronidase (at least three times the dose prescribed in the original solution) [1, 6].

**TABLE 2:** Comparison between original and modified Munhoz‐Cavallieri lavage protocol.

Step	MCP	mMCP
1	Anesthetic button with lidocaine for the needle entry hole	Anesthetic button with lidocaine for the needle entry hole
2	US‐guided drainage was performed using a 21G × 1¼″ or 18G × ½″ needle attached to a 5 or 10 mL empty syringe into the abscess cavity, followed by aspiration for 5 s or until fluid is retrieved. Sterile gel and sterile probe cover were applied to maintain aseptic conditions during the procedure in compliance with the microbiologist recommendation. Needle visualization was performed using the in‐plane technique in all cases, allowing real‐time observation of the entire needle trajectory and confirmation of the needle tip within the collection prior to the initiation of lavage	US‐guided drainage was performed using a 21G × 1¼″ or 18G × ½″ needle attached to a 5 or 10 mL empty syringe into the abscess cavity, followed by aspiration for 5 s or until fluid is retrieved. Sterile gel and sterile probe cover were applied to maintain aseptic conditions during the procedure in compliance with the microbiologist recommendation. Needle visualization was performed using the in‐plane technique in all cases, allowing real‐time observation of the entire needle trajectory and confirmation of the needle tip within the collection prior to the initiation of lavage
3	Solution for lavage protocol: 1500 IU of hyaluronidase diluted with 6 mL of 0.9% sterile saline solution + 1 mL triamcinolone 20 mg/mL + 3 mL of 2% lidocaine for a total of 10 mL of solution	Solution for lavage protocol: 4500–12 000 IU of hyaluronidase diluted with 6–8 mL of 0.9% sterile saline solution +1 mL triamcinolone 20 mg/mL + 1–3 mL of 2% lidocaine for a total of 10 mL of solution
4	Carrying out the washing with a 3 mL syringe and a 22G or 23G needle: injection of 1–3 mL of the solution, aspirating, and disposing. The process is repeated two to three times to wash the collection area. Discard the syringes and needles used in each step	Carrying out the washing with a 3 mL syringe and a 22G or 23G needle: injection of 1–3 mL of the solution, aspirating, and disposing. The process is repeated two to three times to wash the collection area. Discard the syringes and needles used in each step
5	During the third or fourth injection, with sterile syringe and needles, it was deposited 0.5–3 mL of the solution into the cavity, to occupy the purulent secretion space, that is, to collapse the space visualized on the US and dilute the remaining HA	During the third or fourth injection, with sterile syringe and needles, it was deposited 0.5–3 mL of the solution into the cavity, to occupy the purulent secretion space, that is, to collapse the space visualized on the US and dilute the remaining HA

The dose of hyaluronidase is determined by the degree of complication, added to the characteristic and quantity of HA filler used [5].

Severe complications, such as sterile abscess, require high doses of hyaluronidase. HA fillers with lower viscosity, cross‐linking level, molecular weight, and particle size require a lower dose of hyaluronidase, while products with higher levels of 1,4‐butanediol diglycidyl ether (BDDE), viscosity, molecular weight, and particle size (e.g., Volux allergan) may require a higher dose of hyaluronidase for dissolving [5].

Additionally, the quantity, size, and exact location of abscesses also influence the dose determination of hyaluronidase.

As reported by Balassiano, the resolution of sterile abscess caused by more than 2 mL of Juvederm Volux has been challenging during clinical practice and required triple or even quadruple the hyaluronidase dose. Despite that, sterile abscess from 0.5 mL of Juvederm Volux was solved after common doses of hyaluronidase, as demonstrated by Munhoz et al. [1, 6].

The cases reported here received a high volume of high‐rheology HA across multiple facial regions (Table 1). Therefore, quintuple (7500 IU in Case 1) or even more than 20 times the regular hyaluronidase dose (34 875 IU in Case 2) was required to achieve complete resolution of these severe complications.

According to the Complications in Medical Aesthetics Collaborative (CMAC) guidelines, the management of HA complications should involve doses exceeding 1500 IU of hyaluronidase. Furthermore, the guidelines emphasize that, due to the pharmacokinetics of hyaluronidase and its short half‐life following subcutaneous administration (approximately 5.1 min), re‐dosing may be performed 15–20 min after the initial injection to manage the HA‐based vascular occlusion, if necessary [25]. Based on that, and considering the superficial approach of the lavage technique, we propose increasing the hyaluronidase dose per session to three times—or even up to twenty times—the standard dose when treating refractory sterile abscesses. The use of high‐dose hyaluronidase may reduce the number of required lavage sessions, particularly in complications associated with high rheology and large volumes of HA injected in multiple facial regions. This strategy could lead to faster and more favorable clinical outcomes, avoiding prolonged treatment.

CMAC also recommends that, following elective hyaluronidase treatment, patients should be reassessed after 48 h, at which point retreatment may be considered if needed [25]. Although post‐procedural swelling may persist beyond this period, enzymatic activity does not. Therefore, if residual fluid collections are still present on USG after 48 h, the protocol may be safely repeated.

Although the mMCP used doses of hyaluronidase at least three times more than in the label recommended, the cases herewith and previously reported [6] by our group demonstrated no adverse event associated with the high doses of hyaluronidases during and after the treatment.

The main adverse events associated with high doses of hyaluronidase are erythema, edema, pruritus, burning sensation, and bruising at the injection site. Allergy and Type I hypersensitivity reactions are considered rare (0.1%), unless large intravenous doses (in excess of 200 000 IU) have been given, where treatments yielded a Type I reaction rate of 33%. The incidence of type IV reactions is 0.1%. Additionally, the risk of anaphylaxis is extremely rare, with an incidence of less than 0.1% [25, 26].

Wilde et al. had defined the Post‐Hyaluronidase Syndrome, which consists of volume loss, skin depression, loss of skin elasticity, or skin pigmentation changes after the use of hyaluronidase. However, this syndrome occurred in only 18% of cases and was found to be related to the volume and duration of the filler, rather than the dose and concentration of hyaluronidase used [27].

The atrophy reported by Balassiano after mMCP was associated with steroid used in the lavage solution and/or entailed by the healing process related to the intense inflammatory reaction and demonstrated by the outflow of a large quantity of pus [6]. No atrophy or secondary changes were observed beyond the expected local injection site reactions in the cases herewith reported.

DeLorenzi demonstrated that after 2 years of using high‐dose hyaluronidase to treat vascular complications following HA‐filler injections, no long‐term secondary changes were observed beyond the expected local injection site reactions [28].

The cases herewith emphasize the importance of recognizing and correctly managing sterile abscesses to determine a favorable outcome during clinical practice, avoiding prolonged and ineffective treatment of oral antibiotics, corticosteroids, and/or immunosuppressants such as methotrexate and cyclophosphamide.

The inflammatory reaction observed in these cases can be associated with an exacerbated foreign body reaction to HA without the obligatory presence of infection.

The USG was crucial in estimating its total volume, exact layer location, nature, and relationship with the surrounding anatomical structures. USG also allowed the drainage of an abscess under real‐time visualization and its follow‐up until complete resolution.

The risks associated with facial abscess can progress quickly and can be catastrophic to the patient if left untreated, including disfigurement and systemic complications [18, 23]. However, the mMCP has been showing an interesting therapeutic option to a quick resolution of symptoms and a favorable cosmetic outcome.

Despite appearing to be a safe and promising procedure, further studies with larger samples should be carried out to demonstrate the safety and efficacy of the protocol—particularly regarding the use of high‐dose hyaluronidase and the adoption of mMCP as a first‐line treatment for sterile abscesses.

## Author Contributions

All authors have read and approved the final version of the manuscript. Fernanda Cavallieri, Gabriela Munhoz, Laila Klotz de Almeida Balassiano, Anne Kelly Leroy, Maria Fernanda Tembra, Thaissa Bortolato, José Marcos T. Cunha, Marcia Ramos‐e‐Silva, Renata Bregunci Meyer, and Lucia Ribeiro Balsanelli conceptualized, drafted the manuscript, and contributed to the literature and critical revisions.

## Ethics Statement

The authors confirm that the ethical policies of the journal, as noted on the journal's author guidelines page, have been adhered to. The cases reported are sourced from clinical practice. All patients consented to the use of their data and images.

## Conflicts of Interest

The authors declare no conflicts of interest.

## Data Availability

Data sharing not applicable to this article as no datasets were generated.
